# Draft genome sequence of a cyanobacterium *Gloeocapsa* sp. BRSZ, isolated from Bo Khlueng hot spring in Ratchaburi, Thailand

**DOI:** 10.1128/mra.00681-24

**Published:** 2024-10-29

**Authors:** Taiki Aono, Sasiprapa Samsri, Rungaroon Waditee-Sirisattha, Hakuto Kageyama

**Affiliations:** 1 Graduate School of Environmental and Human Sciences, Meijo University, Nagoya, Japan; 2 Department of Microbiology, Faculty of Science, Chulalongkorn University, Bangkok, Thailand; 3 Department of Chemistry, Faculty of Science and Technology, Meijo University, Nagoya, Japan; Rochester Institute of Technology, Rochester, New York, USA

**Keywords:** cyanobacteria, *Gloeocapsa*, mycosporine-like amino acids

## Abstract

We report the draft genome sequence of *Gloeocapsa* sp. BRSZ isolated from Bo Khlueng hot spring in Thailand, comprising 42 contigs assembled at the scaffold level, totaling 6,084,403 bp, with 43.5% G + C content. It contains 5,456 putative protein-coding genes, including genes responsible for biosynthesis of mycosporine-like amino acids.

## ANNOUNCEMENT

Cyanobacteria are promising organisms for the production of useful substances (1). Cyanobacteria inhabit all places on Earth, including extreme environments, and each strain has the potential to synthesize unique secondary metabolites due to specific environmental adaptation mechanisms. The cyanobacterium *Gloeocapsa* sp. Bentong-Raub Suture Zone (BRSZ) was isolated from the Bo Khlueng hot spring (55°C, 13.7368N, 99.2395E) in Thailand, in the BRSZ (2). An axenic culture of *Gloeocapsa* sp. BRSZ was obtained using a micromanipulation technique. The cyanobacterium was cultured in the laboratory in liquid BG-11 medium (3) at 30°C under 12 h light/12 h dark conditions of 70 μE m^−2^ s^−1^. A 1,479 bp region of the 16S rRNA gene of this cyanobacterium was amplified by PCR using the primers 5′-AGAGTTTGATCCTGGCTCA-3′ and 5′-CTAAGGTGATCCAGCCACA-3′, sequenced, and taxonomically identified using the NCBI database. Our analysis revealed that *Gloeocapsa* sp. BRSZ was most closely related (98.85%) to *Gloeocapsa* sp. PCC7428 (GenBank accession number CP003646.1).

Genomic DNA was extracted from *Gloeocapsa* sp. BRSZ using an MPure Bacterial DNA Extraction Kit (MP Bio Japan), following the manufacturer’s instructions. The DNA was quantified using Synergy LX (Agilent Technologies) and the QuantiFluor dsDNA System (Promega). Libraries were prepared using the MGIEasy FS DNA Library Prep Set according to the manual. The quality of the prepared library was confirmed using a Fragment Analyzer and a dsDNA 915 Reagent Kit (Agilent Technologies). Circularized DNA was created using the constructed library and an MGI Easy Circularization Kit (MGI Tech), according to the manual. DNA nanoballs (DNBs) were created using a DNBSEQ-G400RS High-throughput Sequencing Kit (MGI Tech). Paired-end sequencing of 2 × 500 generated DNBs was performed using the DNBSEQ-G400 (MGI Tech). A total of 2,579,904,300 raw reads were obtained, trimmed, and assembled using SPAdes (ver. 3.15.5) (4). The quality of the genome (percentage of completeness of 98.09% and contamination of 5.34%) was assessed using ChekM (ver. 1.2.2) (5). Gene prediction and annotation were performed using NCBI prokaryotic genome annotation pipeline (6). Bioinformatic analyses were performed using default parameters.

The genome sequence of *Gloeocapsa* sp. BRSZ comprised a total of 6,084,403 bp, forming 42 contigs assembled at the scaffold level, with a coverage of 418× and a G + C content of 43.5%. The longest contig was 815,638 bp, with an *N*_50_ value of 552,304 bp. Furthermore, 5,456 putative protein-coding sequences, three rRNA genes, and 40 tRNA genes were identified. Using antiSMASH (ver. 7.1.0) (7), we searched for secondary metabolite biosynthetic gene clusters from the assembled sequences. We identified the presence of mycosporine-like amino acids (MAAs) biosynthetic gene cluster, which included a 2-*epi*-5-*epi*-valiolone synthase, an *O*-methyltransferase, and an ATP-grasp enzyme. We also found gene clusters containing non-ribosomal peptide synthase (NRPS), which could be involved in the biosynthesis of non-ribosomal peptides. NRPS may also be involved in the biosynthesis of MAAs (8). Furthermore, NRPS may be associated with the biosynthesis of polyketides and fatty acids (9). Thus, the *Gloeocapsa* sp. BRSZ genome includes several candidates for a prolific source of biosynthesis of important secondary metabolites.

## Data Availability

The draft genome sequence was deposited in GenBank under the accession number JBEGHC000000000.1; project data are available under BioProject accession number PRJNA1120971, with BioSample accession number SAMN41723176 and SRA accession number SRR29364898. The 16S rRNA gene sequence of *Gloeocapsa* sp. BRSZ has been deposited in NCBI GenBank under the accession number PP907067.
